# Calcium-Sensing Receptor Regulates Cytosolic [Ca^*2+*^] and Plays a Major Role in the Development of Pulmonary Hypertension

**DOI:** 10.3389/fphys.2016.00517

**Published:** 2016-11-04

**Authors:** Kimberly A. Smith, Ramon J. Ayon, Haiyang Tang, Ayako Makino, Jason X.-J. Yuan

**Affiliations:** ^1^Department of Pediatrics, Northwestern UniversityChicago, IL, USA; ^2^Division of Translational and Regenerative Medicine, Department of Medicine, The University of ArizonaTucson, AZ, USA; ^3^Department of Physiology, The University of ArizonaTucson, AZ, USA

**Keywords:** Ca^2+^-sensing receptor, store-operated calcium channel, smooth muscle cells, G-protein coupled receptor, pulmonary artery, pulmonary arterial hypertension

## Abstract

Pulmonary arterial hypertension (PAH) is a progressive disease characterized by elevated pulmonary vascular resistance (PVR) leading to right heart failure and premature death. The increased PVR results in part from pulmonary vascular remodeling and sustained pulmonary vasoconstriction. Excessive pulmonary vascular remodeling stems from increased pulmonary arterial smooth muscle cell (PASMC) proliferation and decreased PASMC apoptosis. A rise in cytosolic free Ca^2+^ concentration ([Ca^2+^]_cyt_) in PASMC is a major trigger for pulmonary vasoconstriction and a key stimulus for PASMC proliferation and migration, both contributing to the development of pulmonary vascular remodeling. PASMC from patients with idiopathic PAH (IPAH) have increased resting [Ca^2+^]_cyt_ and enhanced Ca^2+^ influx. Enhanced Ca^2+^ entry into PASMC due to upregulation of membrane receptors and/or Ca^2+^ channels may contribute to PASMC contraction and proliferation and to pulmonary vasoconstriction and pulmonary vascular remodeling. We have shown that the extracellular Ca^2+^-sensing receptor (CaSR), which is a member of G protein-coupled receptor (GPCR) subfamily C, is upregulated, and the extracellular Ca^2+^-induced increase in [Ca^2+^]_cyt_ is enhanced in PASMC from patients with IPAH in comparison to PASMC from normal subjects. Pharmacologically blockade of CaSR significantly attenuate the development and progression of experimental pulmonary hypertension in animals. Additionally, we have demonstrated that dihydropyridine Ca^2+^ channel blockers (e.g., nifedipine), which are used to treat PAH patients but are only effective in 15–20% of patients, activate CaSR resulting in an increase in [Ca^2+^]_cyt_ in IPAH-PASMC, but not normal PASMC. Our data indicate that CaSR functionally couples with transient receptor potential canonical (TRPC) channels to mediate extracellular Ca^2+^-induced Ca^2+^ influx and increase in [Ca^2+^]_cyt_ in IPAH-PASMC. Upregulated CaSR is necessary for the enhanced extracellular Ca^2+^-induced increase in [Ca^2+^]_cyt_ and the augmented proliferation of PASMC in patients with IPAH. This review will highlight the pathogenic role of CaSR in the development and progression of PAH.

## Introduction

Under normal physiological conditions, the pulmonary circulation is maintained in a high flow, low pressure, and low resistance state. During conditions of increased blood flow or cardiac output (e.g., during exercise), vasodilation and recruitment (opening of closed blood vessels) of pulmonary arteries are two important mechanisms for reducing pulmonary vascular resistance (PVR). However, under pathological conditions, increased PVR is a major cause for the development of pulmonary hypertension. Elevated PVR over time leads to right ventricular hypertrophy and right heart failure.

Pulmonary hypertension is defined by a mean pulmonary arterial pressure (PAP) ≥25 mmHg at rest, as measured by right heart catheterization. Pulmonary arterial hypertension (PAH) describes a subpopulation of patients with pulmonary hypertension, and is hemodynamically defined by a resting mean PAP ≥25 mmHg, pulmonary arterial wedge pressure ≤ 15 mmHg, and PVR >3 Wood units (equivalent to mmHg·min/l) (Hoeper et al., 2013). Under the current clinical classification system established at the fifth World Symposium on Pulmonary Hypertension held in Nice, France in 2013, PAH comprises a group of uncommon conditions characterized by obliterative vasculopathy of the small pulmonary arteries. PAH can be idiopathic (IPAH, when no etiological factors are identified), heritable, induced by drugs or toxins, or be related to conditions, such as connective tissue diseases, congenital heart diseases, portal hypertension, or HIV infection (Simonneau et al., 2013). The population prevalence of PAH is estimated 15–50 cases per million, and the incidence is 2–7 cases per million, making PAH a rare disease (Humbert et al., 2006; Peacock et al., 2007). According to the Registry to Evaluate Early and Long-term PAH Disease Management (REVEAL Registry), an observational US disease registry providing current information about demographics, disease course, and management of patients with PAH, the mean age at diagnosis of PAH is 50.1 ± 14.4 years, with a female to male ratio of 3:1 and a 5-year survival rate from time of diagnosis of 57% (Mcgoon et al., 2008; Badesch et al., 2010; Farber et al., 2015).

## Pathological changes in the pulmonary vasculature in patients and animals with pulmonary hypertension

Although the subcategories of PAH originate from different underlying causes, all forms of PAH can be characterized by a combination of sustained pulmonary vasoconstriction, excessive pulmonary vascular remodeling, *in situ* thrombosis, and arterial wall stiffening (Humbert et al., 2004; Schermuly et al., 2011). Patients with PAH have been shown to have reduced levels of vasodilatory mediators, such as prostaglandin I_2_ and nitric oxide, and increased levels of the potent vasoconstrictors thromboxane, rho-kinase, and endothelin 1 (Christman et al., 1992; Steudel et al., 1997). Additionally, in patients with IPAH and animals with experimental pulmonary hypertension, abnormalities in K^+^ and Ca^2+^ channels have been linked with pathological pulmonary vasoconstriction (Yuan et al., 1998; Yu et al., 2004).

Controlling the balance between cell proliferation and apoptosis of pulmonary arterial fibroblasts, pulmonary arterial smooth muscle cells (PASMC), and pulmonary arterial endothelial cells is essential for maintaining normal structural and functional integrity of the pulmonary vasculature. If the balance is tipped in favor of cell proliferation, thickening of the wall, luminal narrowing, and eventual obliteration can occur. These structural changes that lead to hypertrophy and/or luminal occlusion are referred to as pulmonary vascular remodeling (Yu et al., 2004; Masri et al., 2007). The cellular and molecular mechanisms that lead to vascular remodeling are extremely complex, however it is generally understood that K^+^ channels play a pivot role in this process as they are regulators of vessel tone, cell proliferation and apoptosis. Downregulation of K^+^ channels is linked to sustained depolarization due to enhanced Ca^2+^ entry and diminished K^+^ efflux, which promotes cell proliferation and inhibits apoptosis, respectively. Increased proliferation and hypertrophy of PASMC have been implicated in the development of PAH and these processes, like vasoconstriction, relate in part to disturbed Ca^2+^ homeostasis (Kuhr et al., 2012). We have recently shown that the extracellular Ca^2+^-sensing receptor (CaSR) is upregulated in PASMC and lung tissue from patients with IPAH (Yamamura et al., 2012).

## Characteristics of CaSR as a GPCR

CaSR is a G-protein coupled receptor (GPCR) and a member of family C of the GPCR acids. It has a large extracellular domain that is approximately 600 amino acids long. This large extracellular domain is composed of a bi-lobed Venus-flytrap-like domain which is connected to the seven-transmembrane-domain by a cysteine rich region. The intracellular domain of CaSR contains the COOH domain which is 216 amino acids long (Hendy et al., 2009). The CaSR functions as a dimer in which the venus-flytrap-like domains of each monomer interact. Ca^2+^ binds in the cleft of the venus-flytrap-like domain and causes a conformational change in CaSR which instigates cells signaling events (Hendy et al., 2009). The CaSR couples to G proteins to activate or inhibit multiple intracellular signaling pathways.

CaSR was originally discovered in the parathyroid gland were it maintains Ca^2+^ levels in the blood by adjusting release of parathyroid hormone. When CaSR senses changes in extracellular Ca^2+^ levels, parathyroid chief cells release parathyroid hormone which normalizes Ca^2+^ levels by its actions on kidneys, bones, and indirectly, intestines (Brown et al., 1993; Kurokawa, 1994; Saidak et al., 2009). The primary physiological functions of CaSR are to detect changes in extracellular Ca^2+^ levels, modulate release of parathyroid hormone, and maintain constant blood Ca^2+^ levels. CaSR is widely expressed and mutations in CaSR have been associated with several diseases. Inactivating mutations in CaSR cause familial hypocalciuric hypercalcemia and neonatal severe hyperparathyroidism, while activating mutations cause autosomal dominant hypocalcemia and hypocalcemic hypercalciuria (Hendy et al., 2009).

## CaSR signaling

**Figure 1:**
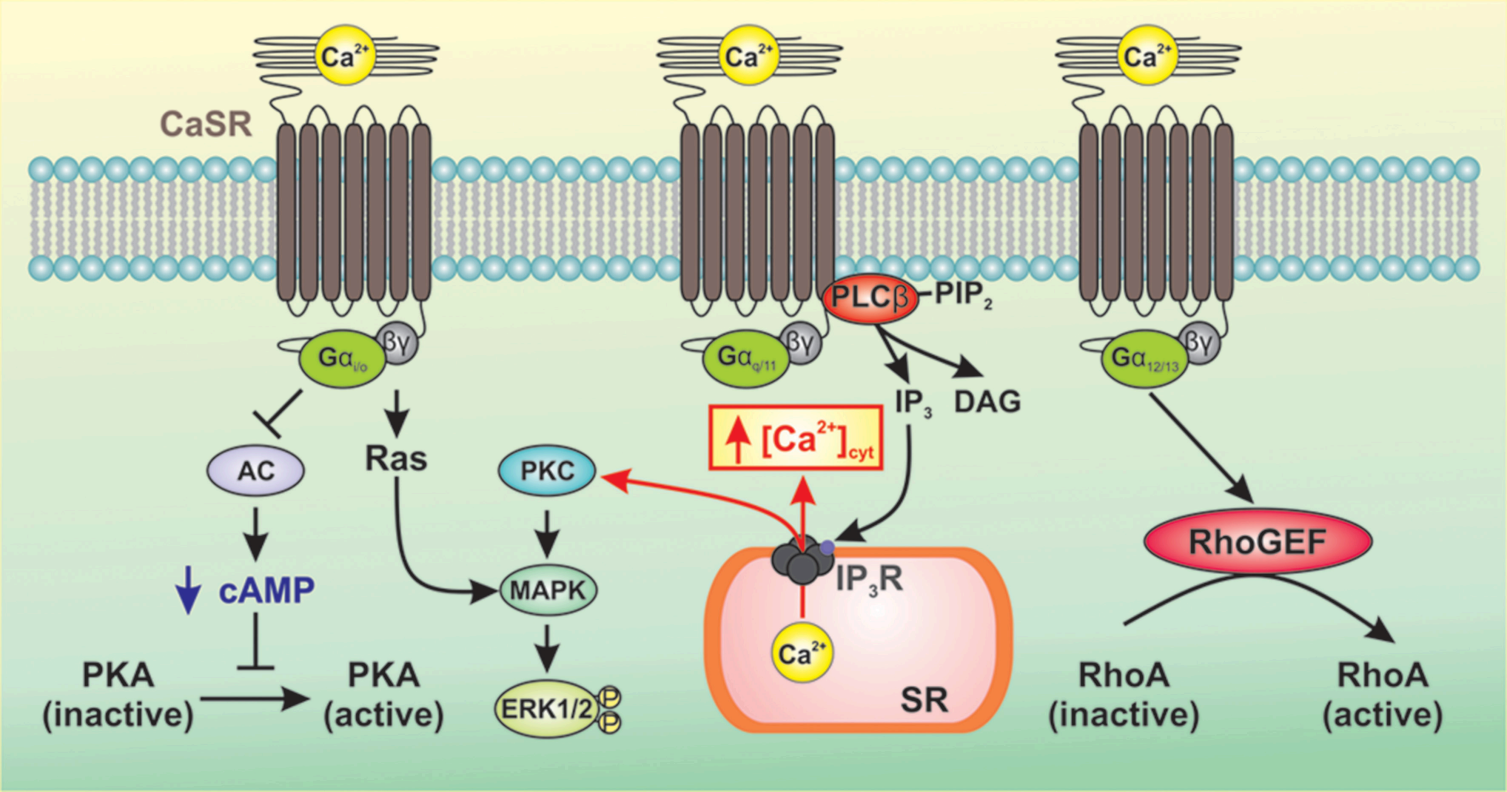
**Generalized representation of CaSR-mediated signaling pathways**. CaSR signals to downstream pathways via three main groups of heterotrimeric G-proteins, G_q/11_, G_i/o_, and G_12/13_. CaSR-mediated activation of G_q/11_ leads to activation of PLCβ resulting in production of IP_3_ which mobilizes cytosolic Ca^2+^ from intracellular Ca^2+^ stores and phosphorylation of PKC, thereby activating MAPK and subsequent phosphorylation and activation of ERK1/2. CaSR-mediated activation of G_i/o_ inhibits AC, which reduces levels of cAMP and PKA activity. The β/γ subunits of G_i/o_ activate Ras leading to MAPK activation and ERK1/2 phosphorylation. Activation of G_12/13_ causes RhoGEF to translocate to the plasma membrane where it activates GEF. RhoGEF then activates RhoA by catalyzing the exchange of GDP for GTP.

As a member of the Class C family of GPCRs, CaSR signals to downstream pathways via three main groups of heterotrimeric G-proteins, G_q/11_, G_i/o_, and G_12/13_ (Conigrave and Ward, 2013; Figure 1). CaSR-mediated activation of G_q/11_ leads to activation of phospholipase Cβ (PLCβ) resulting in conversion of phosphatidylinositol 4,5 bisphosphate to diacylglycerol (DAG) and inositol 1,4,5-triphosphate (IP_3_) (Kifor et al., 2001; Thomsen et al., 2012). This results in an increase in the intracellular Ca^2+^ concentration and phosphorylation of protein kinase C (PKC), which then activates the mitogen-activated protein kinase (MAPK) signaling cascade ultimately resulting in phosphorylation and activation of ERK1/2 (Kifor et al., 2001; Thomsen et al., 2012). CaSR-mediated activation of G_i/o_ results in inhibition of adenylate cyclase (AC) leading to decreased conversion of ATP to cAMP and decreased protein kinase A activity (Kifor et al., 2001; Thomsen et al., 2012). The β/γ subunits of G_i/o_ activate Ras leading to MAPK activation and ERK1/2 phosphorylation. Upon activation of CaSR, the GTP-bound α subunit of G_12/13_ causes RhoGEF to translocate to the plasma membrane and activates its guanine nucleotide exchange factor (GEF) activity. RhoGEF then activates RhoA by catalyzing the exchange of GDP for GTP (Siehler, 2009). Downstream targets of RhoA include ROCK which mediates cell contraction by inhibiting myosin light chain phosphatase activity, resulting in increased myosin light chain phosphorylation. ROCK also plays a critical role in the polymerization and stabilization of actin filaments by direct activation of mDia (mammalian homolog of Drosophila diaphanous) and indirect phosphorylation of the depolymerizing factor cofilin, respectively (Jernigan and Resta, 2014).

**Figure 2:**
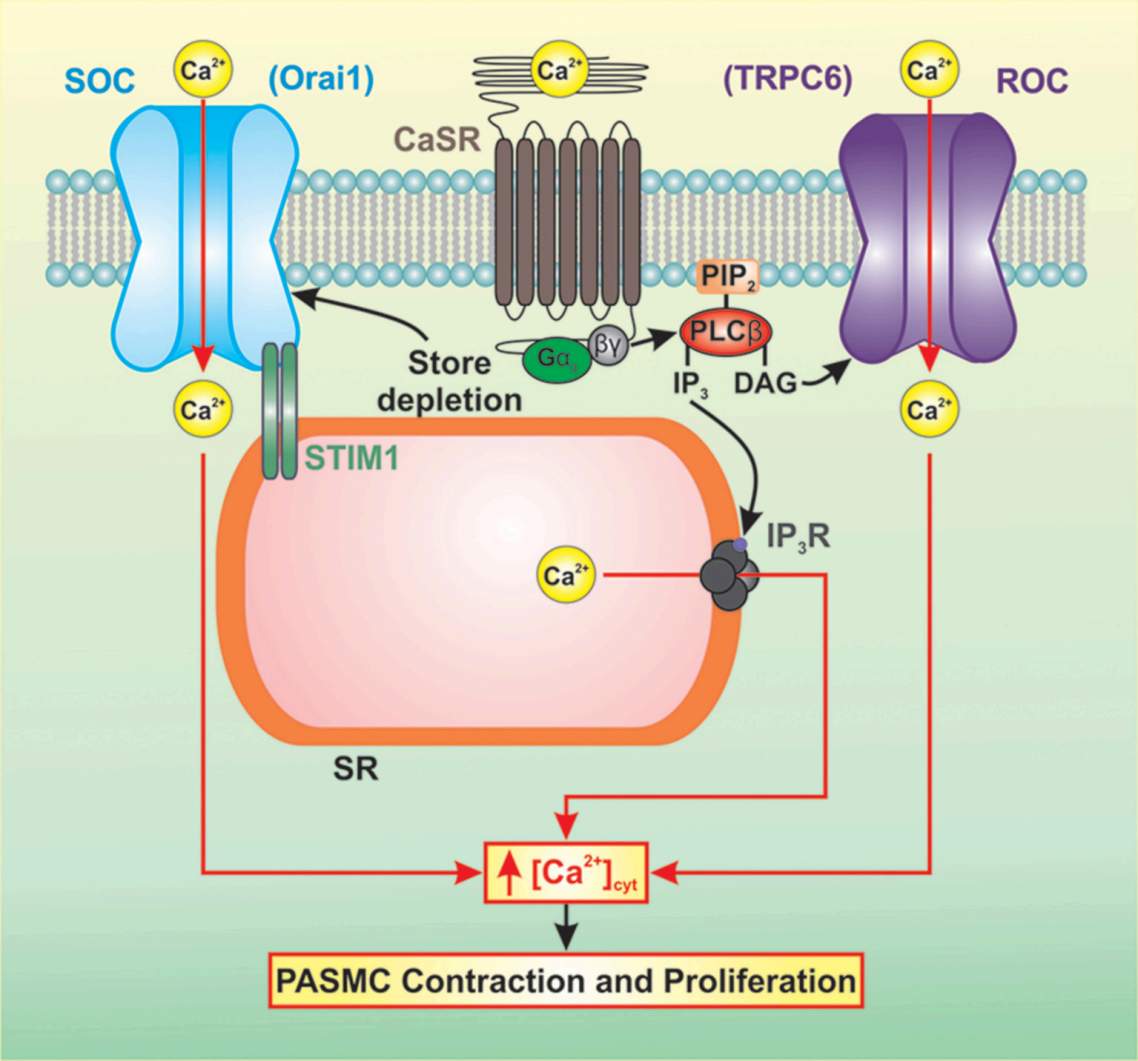
**CaSR-mediated activation of downstream signaling pathways results in increased intracellular Ca^2+^ concentration, increased Ca^2+^ sensitivity, cell contraction, and cell proliferation**. Increased synthesis of IP_3_ and DAG results in elevated [Ca^2+^]_cyt_ via Ca^2+^ release and Ca^2+^ entry. IP_3_ binds to the IP_3_R on the SR releasing Ca^2+^ from the SR to the cytosol. Depletion of Ca^2+^ from the SR induces SOCE through SOCs. DAG activates ROCs in the plasma membrane resulting in ROCE. This rise in [Ca^2+^]_cyt_ in PASMC is a major trigger for pulmonary vasoconstriction and a key stimulus for PASMC proliferation.

CaSR-mediated activation of downstream signaling pathways results in increased intracellular Ca^2+^ concentration, increased Ca^2+^ sensitivity, cell contraction, and cell proliferation (Figure 2). Increased synthesis of IP_3_ and DAG results in the binding of IP_3_ to the IP_3_ receptor (IP_3_R) on the sarcoplasmic reticulum (SR) releasing Ca^2+^ from the SR to the cytosol. In PASMC, depletion of Ca^2+^ from the SR induces store-operated Ca^2+^ entry (SOCE) through store-operated Ca^2+^ channels (SOC). DAG activates receptor-operated Ca^2+^ channels (ROC) in the plasma membrane resulting in receptor-operated Ca^2+^ entry (ROCE). A rise in cytosolic free Ca^2+^ concentration ([Ca^2+^]_cyt_) in PASMC is a major trigger for pulmonary vasoconstriction and a key stimulus for PASMC proliferation. Several studies have demonstrated that resting [Ca^2+^]_cyt_, SOCE, and ROCE are all increased in PASMC isolated from IPAH patients (Yu et al., 2004; Zhang et al., 2007; Song et al., 2011).

## Upregulated expression and enhanced function of CaSR in patients with IPAH and animals with experimental PH

Our studies have demonstrated that application of extracellular Ca^2+^ increased [Ca^2+^]_cyt_ in PASMC isolated from IPAH patients compared to PASMC from control patients and patients with chronic thromboembolic pulmonary hypertension (Yamamura et al., 2012). We found that this is due to increased expression of CaSR in PASMC and lung tissues from patients with IPAH. The extracellular Ca^2+^-induced increase in [Ca^2+^]_cyt_ in IPAH PASMC is dependent on PLC and IP_3_R. Downregulation of CaSR in IPAH PASMC via siRNA reversed the extracellular Ca^2+^-induced increase in [Ca^2+^]_cyt_ and attenuated the enhanced proliferation of IPAH PASMC (Yamamura et al., 2012). Additionally, overexpression of CaSR augmented the extracellular Ca^2+^-induced increase in [Ca^2+^]_cyt_ and enhanced proliferation in normal PASMC (Yamamura et al., 2012). These data demonstrate that increased expression of CaSR and subsequently enhanced CaSR-mediated [Ca^2+^]_cyt_ increase contribute to the enhanced Ca^2+^ signaling and proliferation of PASMC in patients with IPAH.

Using two experimental models of PH, we confirmed the role of CaSR in the development of PH. CaSR mRNA and protein levels are increased in PASMC and small pulmonary arteries in the rat model of monocrotoline-induced pulmonary hypertension (MCT-PH) compared to vehicle injected control rats. Additionally, resting [Ca^2+^]_cyt_ and extracellular Ca^2+^-induced increase in [Ca^2+^]_cyt_ were both enhanced in PASMC from MCT-PH rats, implying that functional upregulation of CaSR in PASMC contributes to the development of pulmonary hypertension in rats injected with MCT (Yamamura et al., 2012). Similar results were seen in the mouse model of hypoxia-induced pulmonary hypertension (HPH). Expression of CaSR mRNA and protein was significantly increased in HPH mice than in normoxic control mice, and extracellular Ca^2+^-induced increase in [Ca^2+^]_cyt_ was significantly enhanced in PASMC isolated from HPH mice (Yamamura et al., 2012). These data suggest that CaSR is functionally upregulated in PASMC from HPH mice. Pharmacological blockade of CaSR with NPS 2143, a CaSR antagonist, significantly attenuated the development of PH in both the rat MCT-PH model and the mouse HPH model. Treatment with NPS 2143 significantly inhibited the increase in right ventricular systolic pressure (RVSP, an indicator of pulmonary arterial pressure) and inhibited the ratio of the weight of the right ventricle to left ventricle plus septum (Fulton's index, an indicator of right ventricular hypertrophy) in MCT-PH rats and HPH mice (Yamamura et al., 2012). Collectively, these data indicate that increased expression of CaSR may play a pathogenic role in the development of PH and that antagonists of CaSR may therapeutic potential for patients with PH.

## CaSR functionally couples to TRPC channels to regulate [Ca^2+^]_cyt_ in patients with IPAH and animals with experimental PH

Upon activation by extracellular stimuli, CaSR induces activation of downstream signaling pathways which result in increased [Ca^2+^]_cyt_. The increased [Ca^2+^]_cyt_ results from Ca^2+^ release from intracellular stores and Ca^2+^ influx through ROC and SOC. Transient receptor potential conical (TRPC) channels have been shown to function as both ROC and SOC in PASMC (Yu et al., 2003, 2004). We recently discovered that CaSR functionally interacts with TRPC6 channels in PASMC from IPAH patients and animals with experimental PH and may play important role in the development and progression of sustained pulmonary vasoconstriction and pulmonary vascular remodeling (Tang et al., 2016). As discussed earlier, extracellular Ca^2+^-induced increase in [Ca^2+^]_cyt_ is enhanced in IPAH-PASMC due to increased expression of CaSR (Yamamura et al., 2012). Blockade of TRPC6 channels significantly inhibits extracellular Ca^2+^-induced increase in [Ca^2+^]_cyt_ in IPAH-PASMC (Tang et al., 2016). Overexpression of CaSR or TPRC6 in normal PASMC results in enhanced extracellular Ca^2+^-induced increase in [Ca^2+^]_cyt_, however, overexpression of both CaSR and TRPC6 dramatically increases extracellular Ca^2+^-induced increase in [Ca^2+^]_cyt_ compared to either alone (Tang et al., 2016). These data strongly suggest that CaSR is functionally coupled to TRPC6 channels in IPAH-PASMC and that receptor—and store-operated Ca^2+^ entry via CaSR-mediated activation of TRPC6 is an important signaling cascade which leads to PASMC contraction, proliferation, and migration in patients with IPAH.

Studies in experimental models of PH revealed that CaSR plays a role in the development of PH. Using CaSR and parathyroid hormone double knockout mice (*casr*^−/−^*/pth*^−/−^; *casr*^−/−^ mice are embryonic lethal) (Ho et al., 1995; Kos et al., 2003), we demonstrated that deletion of CaSR inhibited the development of PH in mice exposed to chronic hypoxia. Exposure of *casr*^−/−^ mice (i.e., *casr*^−/−^*/pth*^−/−^ mice) to chronic hypoxia results in attenuated RVSP, Fulton's Index, and pulmonary arterial wall thickness compared to chronically hypoxic wildtype mice (Tang et al., 2016).

## Target CaSR to develop novel therapeutic approach for PAH

Calcium channel blockers (CCBs) have been successfully administered as a long term therapy to a number of PAH patients who respond positively to acute vasodilator challenge (Puri et al., 2007). Unfortunately only 10–15% of PAH patients actually meet the criteria, and within this subgroup only about half the patients exhibit any sustained benefit from using CCBs (Sitbon et al., 2005). Due to advances in the understanding of the pathological mechanisms of PAH, drug therapy for PAH has progressed in recent years via the development of several specific drugs that offer an effective alternative to CCBs, such as nifedipine and diltiazem. Most recently, efforts have turned toward the use of CaSR antagonists, also known as calcilytics, as potential drug candidates for treatment of PAH. Two structurally distinct calcilytics NPS2143 and Calhex 231 were shown to suppress excessive cell proliferation of IPAH-PASMCs, but not in normal or CTEPH (chronic thromboembolic pulmonary hypertension) PASMCs, whereas R568, an activator of CaSR, significantly enhanced the proliferation of IPAH-PASMCs (Yamamura et al., 2015). Additionally, NPS2143 but not R568 was shown to attenuate, the extracellular Ca^2+^-induced [Ca^2+^]_cyt_ rise in IPAH-PASMC. Furthermore, intraperitoneal injection of NPS2143 prevented the development of pulmonary hypertension and right ventricular hypertrophy in MCT-PH rats and CH-PH mice (Yamamura et al., 2012). Phosphodiesterase type 5 (PDE5) inhibitors are widely used to treat IPAH patients. Sildenafil inhibits excessive cell proliferation of IPAH-PASMC, but does not affect the cell growth of PASMC from normal subjects and CTEPH patients. In combination with NPS2143 or Calhex 231, the antiproliferative effect induced by sildenafil was significantly enhanced in IPAH-PASMC (Yamamura et al., 2016). These findings reveal that CaSR antagonists and PDE5 inhibitors work together to additively suppress the excessive cell proliferation of IPAH-PASMC, suggesting that a combination therapy of a PDE5 inhibitor with a calcilytic may be useful as a novel therapeutic approach for IPAH.

## Summary and conclusion

An atypical rise in [Ca^2+^]_cyt_ is a major cause for sustained pulmonary vasoconstriction, and excessive pulmonary vascular remodeling observed in patients of IPAH. Our group has previously shown that expression of CaSR, a member of the GPCR family, is upregulated in PASMC isolated from IPAH patients and in animal models of pulmonary hypertension. Additionally, CaSR has been shown to be necessary for the increased extracellular Ca^2+^ influx and subsequent elevation of [Ca^2+^]_cyt_, that in turn leads to enhancement of cellular proliferation, contraction, and migration in IPAH-PASMC. Therefore, it is feasible to say that upregulated CaSR in PASMC presents a novel pathogenic mechanism contributing to sustained vasoconstriction and excessive vascular remodeling seen in IPAH patients. Our group has also shown that CaSR regulates ROCE and SOCE via activation of TRPC6 in IPAH-PASMC from patients and animals with experimental pulmonary hypertension suggesting a functional coupling between CaSR and TRPC6. Pharmacological blockade and targeted deletion of CaSR has been shown to inhibit the CaSR-mediated increase in [Ca^2+^]_cyt_ and attenuates the development of experimental-induced pulmonary hypertension in animal models of the disease. Thus, pharmacological targets of CaSR may reveal novel therapeutic strategies for controlling aberrant Ca^2+^ signaling observed in PAH patients.

## Future research direction

While it has been demonstrated that both CaSR and TRPC6 are upregulated in PASMC from IPAH patients and animals with experimental pulmonary hypertension and play a critical role in the pathogenesis of PAH, it remains unknown how this is achieved. The human CaSR gene, CASR, can be transcriptionally regulated by transcription factors, post-transcriptionally regulated by microRNAs and epigenetically regulated by DNA methylation (Hendy et al., 2009). The promoter region of CASR contains numerous binding sites of transcription factors that are associated with cell proliferation (Fantozzi et al., 2003; Firth et al., 2010; Crosswhite and Sun, 2014). Not surprisingly, several of these transcription factors have been demonstrated to be upregulated in PASMC from patients with IPAH and animals with experimental PH (Yu et al., 2003; Firth et al., 2010; Crosswhite and Sun, 2014; Zabini et al., 2015). It is likely these upregulated transcription factors bind directly to the promoter of CASR and activate CASR transcription. MicroRNAs (miRNA or miR) are another area of interest as they can potentially regulate the CaSR mRNA and protein level by directly binding to the 3′-UTR of CaSR mRNA thereby decreasing expression levels via inhibition of protein translation or increase the rate of mRNA degradation. Using the *in silico* prediction approach (provided by microrna.org), we have identified several miRNAs that may potentially target CASR. Further efforts should focus on *in vitro* and *in vivo* approaches to examine the potential involvement of upregulated/downregulated miRNAs in the upregulation of CASR shown in PASMC from IPAH patients and animals with experimental PH.

## Author contributions

KS: Drafted Manuscript. KS, RA, HT, AM, JY: Edited Manuscript. KS, RA, HT, AM, JY: Approved Final Submission.

## Funding

This work was supported in part by grants from the National Heart, Lung, and Blood Institute of the National Institutes of Health (HL-115014, HL-066012, HL-125208).

### Conflict of interest statement

The authors declare that the research was conducted in the absence of any commercial or financial relationships that could be construed as a potential conflict of interest.
